# *POLE*-mutated endometrial cancer: new perspectives on the horizon?

**DOI:** 10.3389/fonc.2025.1633260

**Published:** 2025-08-27

**Authors:** Daniele Fanale, Lidia Rita Corsini, Paola Piraino, Erika Pedone, Chiara Brando, Tancredi Didier Bazan Russo, Pietro Ferraro, Alisia Simone, Silvia Contino, Ornella Prestifilippo, Ugo Randazzo, Ambra Giurintano, Carla Ferrante Bannera, Antonio Galvano, Lorena Incorvaia, Gianfranco Pernice, Salvatore Vieni, Gianni Pantuso, Calogero Cipolla, Antonino Giulio Giannone, Giuseppe Badalamenti, Antonio Russo, Viviana Bazan

**Affiliations:** ^1^ Section of Medical Oncology, Department of Precision Medicine in Medical, Surgical and Critical Care (Me.Pre.C.C.), University of Palermo, Palermo, Italy; ^2^ U.O. Oncologia, Fondazione Istituto G. Giglio, Palermo, Italy; ^3^ Division of General and Oncological Surgery, Department of Precision Medicine in Medical, Surgical and Critical Care (Me.Pre.C.C.), University of Palermo, Palermo, Italy; ^4^ Pathology Unit, Department of Health Promotion Sciences, Maternal and Infant Care, Internal Medicine and Medical Specialties (PROMISE), University of Palermo, Palermo, Italy; ^5^ Department of Biomedicine, Neuroscience and Advanced Diagnostics, University of Palermo, Palermo, Italy

**Keywords:** endometrial cancer, exonuclease domain mutations, germline/somatic *POLE* variants, microsatellite instability, *POLE*, *POLE*-ultramutated subtype, prognosis

## Abstract

Endometrial carcinoma (EC) is one of the most common gynecological cancers showing a survival rate of 15-17% in the case of advanced disease. Based on the mutational burden and copy number alteration, EC is classified into four different molecular subgroups: *POLE*-mutated (ultramutated), microsatellite unstable (hypermutated), low copy number (endometrioid), and high copy number (serous-like). Despite the high tumor grading, the ultramutated subtype, accounting for about 8-10% of all ECs, showed favorable prognostic potential, enhanced immune response, and excellent clinical outcomes. Somatic POLE alterations have been found in 6-10% of ECs, whereas germline pathogenic variants have been reported only in 0.25-4% of cases. Germline *POLE* alterations are linked to genome instability and are associated with onset of hereditary tumors, including colorectal cancer and EC. Emerging data suggests that knowledge of *POLE* mutational status could be clinically important, as ultramutated ECs may be more likely to respond to immunotherapy. In this Review, we will investigate the role of germline/somatic *POLE* genetic alterations in EC, discussing the potential future theranostic applications and evaluating the benefit of performing a routine genetic testing, in order to adopt prevention and surveillance strategies in germline *POLE* mutation carriers.

## 1 Introduction

Endometrial carcinoma (EC) is one of the most common gynecological cancers with a steady increase in incidence worldwide, accounting for 6th most frequent cancer in women (1, 2). Approximately 382,000 new cases of EC and 90,000 deaths are diagnosed annually worldwide (2). In particular, the high EC incidence rate in North America and Western Europe may be associated with a high presence of lifestyle-related risk factors, such as obesity (3). Racial disparity and socioeconomic and geographical differences are important variables for EC-related incidence and mortality (4). Although most women show at an early-stage disease favorable prognosis, with 5-year overall survival (OS) of 81%, some patients present with advanced disease, and the 5-year OS for stages IVA and IVB is only 17% and 15%, respectively (5, 6).

EC may be classified based on main three aspects: pathogenetic, histopathological and molecular (7). In 1983, Bokhman (8) described two pathogenetic types of EC: type I, more common, favorable from a prognostic point of view, associated with obesity, metabolic syndrome and hyperestrogenism, and type II, unrelated to risk factors known at that time (9).

The histopathological classification of EC includes several forms: endometrioid and its variants, mucinous, serous, clear cell, neuroendocrine, mixed, undifferentiated, or dedifferentiated. In addition, there are also some carcinomas with mesenchymal differentiation, defined as carcinosarcomas (10). It is possible to highlight a correlation between the histopathological subtypes and pathogenetic types: type I tumors show an endometrioid histology in 70%-80% of cases; type II tumors may have serous, clear cell, or undifferentiated histology and are more clinically aggressive (10, 11).

**Figure 1:**
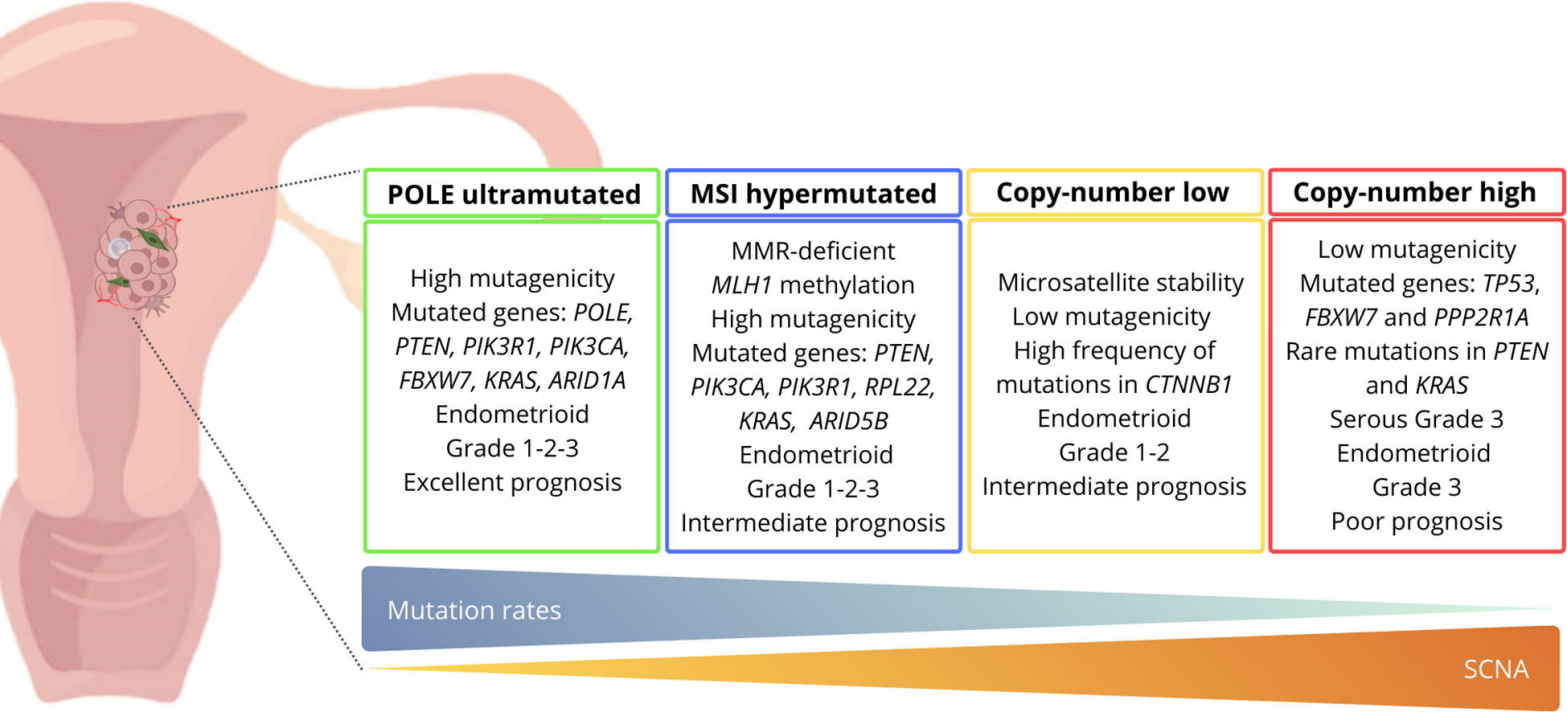
Molecular classification of endometrial cancer and related characteristics according to the TCGA criteria. *ARID1A*, AT-rich interaction domain 1A; *ARID5B*, AT-rich interaction domain 5B; *CTNNB1*, Catenin beta-1; *FBXW7*, F-box/WD repeat-containing protein 7; *KRAS*, Kirsten rat sarcoma viral oncogene homolog; *MLH1*, mutL homolog 1; MMR, mismatch repair; MSI, microsatellite instability; *PIK3CA*, phosphatidylinositol-4,5-bisphosphate 3-kinase; *PIK3R1*, phosphoinositide-3-kinase regulatory subunit 1; *POLE*, DNA polymerase epsilon, catalytic subunit; *PPP2R1A*, protein phosphatase 2 scaffold subunit Aalpha; *PTEN*, phosphatase and tensin homolog; *RPL22*, ribosomal protein L22; SCNA, somatic copy number alterations; *TP53*, cellular tumor antigen 53.

For the first time, the Cancer Genome Atlas (TCGA), through a comprehensive genomic analysis, classified EC into molecular subgroups based on the mutational burden and copy number alterations (12). The updated classification allowed to identify four distinct subgroups: *POLE*-mutated (ultramutated), microsatellite unstable (hypermutated), low copy number (endometrioid), and high copy number (serous-like) (11, 13, 14) (**Figure 1**). Generally, endometrioid carcinomas occur in all molecular subgroups, instead serous carcinomas arise almost exclusively in the high copy number subgroup (10).

Based on the TCGA classification, the Proactive Molecular Risk Classifier for Endometrial Cancer (ProMisE) used surrogate biomarkers to summarize the TCGA subtypes, in order to evaluate the utility of those classifiers in risk prediction (14).

Like ProMisE, Stelloo et al. (15), by means of subsequent immunohistochemistry (IHC) studies, classified EC into four subgroups: POLE-mutated, microsatellite instability (MSI) positive, p53 mutant, and no specific molecular profile (NSMP). The *POLE*-mutated and MSI-positive subgroups showed the most favorable outcomes (6, 10).

The use of these new integrated ‘histomolecular’ diagnostic entities offers the possibility to characterize EC in a different way and improve the risk assessment and treatment in patients, adding interesting diagnostic, prognostic, and therapeutic objectives (15, 16). The POLE-ultramutated subgroup is very promising as regards the perspectives of EC women (10, 11, 14).

Several studies have investigated the clinical impact of the detection of a POLE mutation in EC. Levine et al. (12), in a study published in 2013, found possible correlations between the presence of POLE alterations and therapeutic applications. Since then, much more attention has been paid to the prognostic and predictive role of POLE mutations in EC, as highlighted by the investigation by León‐Castillo et al. (17). Finally, the RUBY study provided important evidence on the role of POLE mutations in EC and their impact on the response to immunotherapy (18). All these studies, together with others, have allowed a better understanding of the biology of EC, providing an important contribution to risk stratification.

Therefore, in this Review, we will investigate the role of germline *POLE* genetic alterations in EC predisposition, discussing the potential future theranostic applications and evaluating the benefit of performing a routine genetic testing, in order to adopt prevention and surveillance strategies in patients harboring germline *POLE* pathogenic variants (PVs) and unaffected family members.

## 2 *POLE*: more than believed

### 2.1 *POLE* functions

POLE is involved in DNA replication process and has been recently recognized as an inherited gene able to predispose to cancer (19). POLE alterations are linked to genome instability and are associated with the prognosis and development and onset of tumors, including colorectal cancer (CRC) and EC, which results in an “ultramutated” phenotype (13, 20).

*POLE*, also known as *POLE1*, *FILS* and *CRCS12*, is located on the human chromosome 12q24.3. Its gene product, the largest subunit of Pol ϵ, contains 2286 amino acids and has a molecular weight of 262 kDa (21).

**Figure 2:**
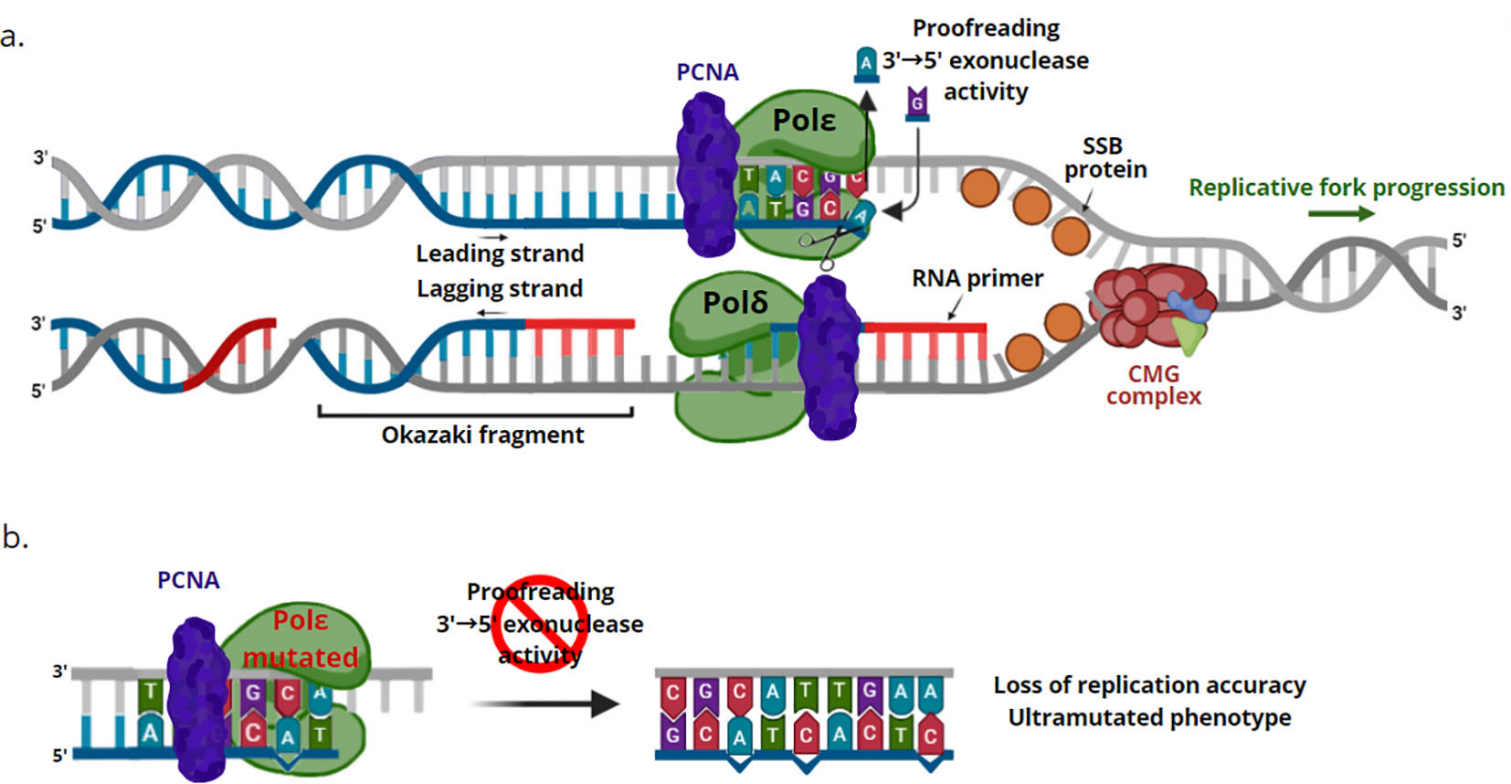
Functioning mechanism of DNA polymerase ϵ. **(a)** Proofreading activity. The neosynthesized DNA strand is indicated in blue, while the RNA primers are represented in red. Polymerase ϵ synthesizes the guide strand, continuously following the progression of the replicative fork. Polymerase δ uses the other strand as a template, moving in the opposite direction from the replicative fork, generating the lagging strand. The resulting Okazaki fragments contain around 200 nucleotides in human cells; for convenience, they are shown shorter in the figure. When an incorrect nucleotide is incorporated, the 3’ end of the newly synthesized strand moves from the polymerase active site to the 3’-5’ exonuclease domain (exonuclease proofreading). The incorrect nucleotide is removed, allowing the formation of the primer-template junction, which can bind the polymerase active site, permitting DNA synthesis to continue. **(b)** Pol ϵ deficiency. Genetic alterations which inactivate or suppress the exonuclease proofreading activity of the polymerase ϵ cause increased replicative error rates and an ultramutated phenotype. CMG complex, Cdc45-Mcm2-7-GINS complex; PCNA, proliferating cell nuclear antigen; POL δ, DNA polymerase δ; POL ϵ, DNA polymerase ϵ; SSB protein, single-stranded binding protein.

The *POLE* gene encodes the main catalytic subunit of the enzyme DNA polymerase ϵ, which synthesizes leading and lagging strands, during DNA replication in eukaryotes. Pol ϵ and Pol δ both belong to the DNA polymerase B family, which have polymerase and 3′-5′ exonuclease activities. Polymerase ϵ is believed to work in leading strand synthesis, although there is conflicting data on whether polymerase δ synthesizes both the leading and lagging strands, and that polymerase ϵ serves only in repair and proofreading activity (22, 23). The proofreading activity allows polymerase ϵ to recognize and remove incorrect nucleotides through its exonuclease activity, thus enabling high-fidelity DNA replication (24) (**Figure 2a**).

*POLE* alterations can occur in the polymerase exonuclease domain, within hotspot regions. These genetic alterations inactivate or suppress the 3′ to 5′ proofreading capabilities of the polymerase, causing a loss of replication fidelity, the occurrence of genomic instability, an increase in replicative error rates and consequently an ultramutated phenotype (11, 22) (**Figure 2b**).

The effects of individual mutations on exonuclease activity are likely to be distinct. Some mutations in exon 13, which hosts the exonuclease domain, can generate more than 8,000 neo-epitopes, favoring and determining a highly immunogenic microenvironment, as highlighted by an increase in the number of peri-tumor and tumor-infiltrating lymphocytes (TILs) and by a concomitant high expression of PD-1 or PD-L1 (25). Indeed, TILs express the PD-1 receptor, playing an important role in tumor immune escape (26). Also, *POLE*-mutated tumors demonstrated a high frequency of transversions from C to A (22). The presence of germline *POLE* PVs is a predisposing factor for EC and CRC, but also for ovarian cancer and brain tumors (21).

### 2.2 *POLE* alterations

The Cancer Genome Atlas (TCGA) project first allowed to identify the molecular subset of POLE-ultramutated EC, characterized by a favorable prognostic potential, enhanced immune response, and excellent clinical outcomes, despite the high tumor grading (11). The *POLE*-ultramutated subtype, accounting for about 8-10% of all ECs, is characterized by *epsilon polymerase* (POLE) exonuclease domain mutations (EDMs) with extremely high mutation rates (≥100 mutations/Mb) (19, 20). In particular, the pathogenicity of five most common *POLE* variants (P286R, V411L, S297F, A456P and S459F) has been shown. The five *POLE* mutation hotspots were associated with an elevated tumor mutational burden (TMB = 268 mut/Mb), which varied between different mutational hotspots (range 37.5-791.9 mut/Mb) and between tumors with identical hotspot mutations (e.g. P286R: 41.9-550.1 mut/Mb). *POLE* hotspot mutant ECs typically exhibited a high rate of C>A and T>G substitutions, whereas the percentage of C>G and indel substitutions were low (17).

A recent work by León‐Castillo et al. (17) showed that POLE-mutated ECs typically exhibit specific genomic alterations: a high prevalence of C>A substitutions (often greater than 20%), a low percentage of small insertion and deletion mutations (indels), and a very high TMB (>100 mut/Mb). In a study performed by the TCGA, 17 cancers classified as ultramutated had a POLE EDM, including the most frequent P286R (exon 9) and V411L (exon 13) substitutions (eight and five cases, respectively) and one case each of M444K, A456P, L424I, and S297F substitutions. Interestingly, 10 out of the 231 non-ultramutated ECs also harbored a *POLE* variant inside or outside the exonuclease domain (12).

In addition to the TCGA study, further investigations confirmed the prevalence of the five PVs and identified additional alterations having uncertain pathogenicity. In their study, Leòn-Castillo and colleagues developed a scoring system able to assess the pathogenicity of new *POLE* variants, on the basis of the presence or absence of genomic alterations associated with known *POLE* PVs. This scoring system was improved, assessing whether *POLE* alterations were recurrent in EC within the COSMIC or TCGA databases (17), since recurrent mutations are more likely to be pathogenic as they are responsible for tumor ultramutation (27). Therefore, the presence of “recurrence” was incorporated into the final scoring system related to pathogenicity, called the “POLE-score”. The “POLE-score” system allows to predict the pathogenicity of *POLE* mutations in EC, when its value is ≥ 4 points. Instead, POLE EDMs in EC with a “POLE score” ≤ 2 were classified as non-pathogenic, based on the absence of genomic alterations associated with the ultramutated phenotype. Finally, tumors with a score of 3 were classified as having a variant of uncertain significance (e.g., L424V, T278M, A428T and A465V). Furthermore, pragmatic guidelines for interpreting the clinical significance of *POLE* variants, where no complete genomic data is available, were provided, in order to evaluate the prognostic and therapeutic value associated with ultramutated-POLE ECs. The EC mutant subtype harboring POLE EDMs attracted more attention due to better results obtained in terms of survival (17).

#### 2.2.1 Technique approaches for genetic testing

The most frequent somatic alterations detected in ECs, within the POLE proofreading domain, are P286R, V411L, and S459F, which have been identified in 67-92% of POLE-mutated tumors. The recommendations of the revised WHO classification system and ESMO guidelines for EC suggest to test the five *POLE* “hotspot” mutations by means of the Next-Generation Sequencing (NGS) and Sanger sequencing (28). In the work by Leòn Castillo et al. (17), these five hotspot PVs in the *POLE* gene were detected, with a frequency of 10.8%, in Formalin-Fixed Paraffin-Embedded (FFPE) samples from EC women, using droplet digital PCR (ddPCR). Approximately 30% of *POLE* variants identified by ddPCR were not detected by Sanger sequencing. The *POLE* mutations with low allele frequency are likely be undetected, due to the limit of detection of Sanger sequencing. Interestingly, the most common alterations detected by ddPCR were V411L (67%) and P286R (25%), whereas TCGA data analysis revealed that P286R was more common than V411L (17). Using ddPCR to detect POLE hotspot mutations could integrate the current ProMisE classification system, which involves immunohistochemistry to evaluate MMR status, followed by *POLE* genetic testing and immunohistochemistry analysis to evaluate the expression of p53 (29). Furthermore, Murali et al. (30) also suggested that *POLE* sequencing should be performed before immunohistochemistry evaluation for MMR status, since the expression loss for MMR proteins in a *POLE*-mutated case would be assigned to the dMMR (MMR deficiency) subgroup rather than *POLE*-positive subgroup. Some researchers suggested to perform the immunohistochemistry analysis and ddPCR simultaneously to evaluate the status of POLE, MMR, and p53, which could prevent misclassification and then be used for prognosis and treatment choice (17). Another innovative genetic analysis is represented by the OncoPanel test, reported in study by Veneris et al. (22). The OncoPanel test, developed at the Dana-Farber Cancer Institute, is a targeted NGS test, which, in addition to detecting somatic alterations, also allows to identify copy number variation and structural DNA rearrangements, and to perform mutational signature analysis. The current version of the OncoPanel test analyzes exon sequences of 447 genes involved in cancer biology and 191 regions of 60 genes for the rearrangement detection (22).

Mutational signature analysis is a unique aspect of this panel. Mutational signatures which can be detected with this approach include DNA damage patterns associated with exposure to UV light, tobacco and alkylating agent, and with apolipoprotein mRNA editing enzyme dysregulation B (APOBEC), MMR deficiency and impairment of *POLE* function. Each carcinogen or DNA repair defect leaves a characteristic “imprint” describing the mode in which DNA is altered. The *POLE* signature shows a strand distortion for C-to-A transitions at TpCpT positions and T-to-G mutations in the TpTpT genomic context. Currently, 30 mutational signatures have been characterized, the list of which it is possible to find in the Catalog of Somatic Mutations in Cancer (COSMIC) database. A specimen is considered to harbor a mutational signature if it is detected, so it is possible for a sample to be attributed with more than one (or no) signature (22).

### 2.3 *POLE/TP53* correlations

The presence of secondary *TP53* alterations has been reported in up to 42% of *POLE*-mutated EC cases (16). In the study carried out by Vermij et al. (16) a small subgroup of patients (3-5%) showed more than one classifying alteration (e.g., POLE-mut/dMMR EC, POLE-mut/p53abn EC, dMMR/p53abn EC or POLE-mut/dMMR-p53abn EC), which allowed to define these ECs as *“multiple classifiers”*.

Certainly, the combination of POLE-mut/p53abn EC is the most controversial, as the tumor harbors a favorable PV in the POLE domain of the exonuclease but also an unfavorable aberrant IHC expression of p53. In opposition to the excellent prognosis of POLE-mutated ECs, p53abn ECs are associated with poor clinical outcomes (16).

Molecular clustering of these “multiple classifier ECs” showed that POLE-mut/p53abn ECs clustered together with POLE-mut ECs without *TP53* alterations. In addition, it was observed that p53-IHC, in these cases, often showed “subclonal” expression of mutant-like p53. This unusual expression pattern of p53 can be observed in POLE-mut and dMMR ECs, reflecting their genetic heterogeneity. Available survival data demonstrated that POLE-mut/p53abn ECs exhibit clinical outcomes comparable to POLE-mut EC without abnormal p53 expression. This would appear to indicate that *TP53* mutations in these “multiple classifiers” are likely transient and do not affect clinical behavior, indicating that these cases should be classified and treated as POLE-mut ECs (16).

### 2.4 POLE/MSI correlations

POLE-mut ECs have been shown to be genetically distinct from MSI-high tumors (20). The most common alteration in MSI-H tumors is the frameshift/deletion mutation, whereas the most frequent variant in POLE-mutated tumors is the missense mutation. Tumors with dMMR/MSI-H or *POLE* mutations are usually associated with high TMB. MSI-H is mainly restricted to tumors in the 10–100 mut/Mb range, whereas TMB of *POLE*/*POLD1* alterations can overcome 100 mut/Mb. *POLE* mutation and MSI were classified as ultra-high mutation phenotype and strong mutation phenotype, respectively (21).

Mismatch repair deficiency combined with loss of proofreading activity of the replication polymerase can produce DNA repair defects, resulting in an ultrahigh mutation with microsatellite stability (MSS) (31). TCGA considers POLE mutation and MSI as two important indicators of molecular typing. However, up to 30% of *POLE*-mutated tumors exhibit MMR deficiency or elevated MSI (21). Since universal screening for Lynch syndrome using MMR IHC has been widely used (32), MMR-deficient POLE EDM ECs may represent a confounding factor in the screening process (20). *POLE* mutations coexisting with MSI in EC are more likely to be non-exonuclease and non-pathogenic, although this is not an universal assumption. A pragmatic tool was developed in order to classify EC with somatic *POLE* mutations in clinical practice (17). The POLE score and presence or absence of MSI/dMMR can be used to stratify EC cases in POLE-mut, dMMR, or one of the other two TCGA subgroups depending on p53 status (33). The only presence of a POLE deleterious alteration is not sufficient to categorize tumors as “POLE-mut”, but the classification of ECs with combined POLE alteration and dMMR/MSI depends on the POLE score (17, 22).

### 2.5 Prognostic value of POLE in EC

POLE-mut ECs are characterized by a favorable prognosis. Several hypotheses have been proposed about the prognosis of POLE-mut ECs, the main one being that the extremely high mutational load may generate a major immune response (34). A less favored hypothesis is that intrinsically defective DNA repair related to POLE mutations makes tumor cells more susceptible to standard chemotherapy, although *in vitro* data showed platinum resistance rather than susceptibility. Another little-established hypothesis is that the abundance of mutations may result deleterious for cancer cells, reducing the their ability to proliferate or metastasize. However POLE-mutated tumors appear as widespread diseases, at high risk of relapse (22).

Li et al. (35) demonstrated that POLE mutations improve the prognosis of EC by regulating cellular glucose metabolism via AMF/AMFR signal transduction pathway.

On the one hand, the POLE PVs greatly affect patient prognosis, on the other hand, 3-6% of EC patients exhibit multiple molecular alterations, including POLE/p53 mutations and dMMR. These patients usually have a good prognosis, if they are carriers of a POLE PV (36).

An emerging link has been observed between high TMB and improved prognosis in cancer patients (37). Indeed, POLE-mut ECs exhibit higher immune infiltration and PD-1/PD-L1 expression, which may compensate for low survival risk caused by higher tumor grades in ultramutated POLE tumors, determining a favorable prognosis (38).

In 2020, the ESGO/ESTRO/ESP published their joint guidelines for the management of EC patients, incorporating findings of the TCGA to assess the prognosis in association with conventional and distinct clinic-pathological prognostic factors (tumor stage, grade and histotype, myometrial invasion or lymphovascular space invasion) in risk stratification of EC (39). However, several points remain to be clarified, since the prognostic value of the TCGA molecular group may vary depending on different EC histotypes (19).

## 3 Therapeutic approaches in POLE-mutated EC

### 3.1 POLE and therapy

The standard treatment for EC is surgery involving total hysterectomy and bilateral salpingo-oophorectomy, with lymph node evaluation (40). Adjuvant treatment often involves significant side effects and toxicities. Therefore, the objective of oncologists is to find an optimal patient selection approach, in order to reduce recurrence risk, improve survival, and avoid overtreatment side effects (41). The clinical and surgical histopathological features, which stratify patients into low-risk, low-intermediate, high-intermediate and high-risk groups, determine the need and type of adjuvant treatment (6, 42). Molecular classification allowed to classify EC into four subgroups: POLE-mutated, p53-abnormal, dMMR, and NSMP (no specific molecular profile). These four groups of ECs represent a promising clinical tool to assist in decision making regarding adjuvant treatment (43). Specifically, the POLE mutations are associated with a low relapse risk, probably due to the high mutational burden of highly immunogenic POLE-mutated ECs (44). This setting of patients will likely not require any adjuvant treatment in the early stages. Conversely, patients harboring p53-altered tumors have a high risk of recurrence and reduced survival, therefore require a chemotherapy treatment (6, 45).

When the molecular classification is known, according to the new guidelines of The European Society of Gynaecological Oncology (ESGO), adjuvant treatment is not recommended in stage I-II patients harboring POLE alteration, due to lack of benefit (7). Furthermore, advanced stage POLE-mutated patients (III-IVA) are not included in this category by the ESGO guidelines, due to data restrictions. These guidelines also suggest not performing the POLE mutational analysis for low-risk patients. Indeed, for patients who do not require adjuvant treatment based on clinic-pathological parameters, the knowledge of the POLE mutational status would has no impact on clinical management (6, 39).

Clinical trials, such as PORTEC-4a, RAINBO, CANSTAMP and TAPER, are playing a key role in the development of personalized treatments against EC, based on molecular and clinic-pathological features, leading to more tailored care strategies for patients (**Table 1**) (6, 50, 52, 53).

**Table 1:** Clinical trials regarding personalized treatments against EC based on molecular and clinic-pathological features.

Trial name	Phase	Design	Patient population	Intervention arms	Primary endpoints	References
PORTEC-4a	III	Multicenter, International, randomized trial (2:1)	550 women ≥ 18 years high-intermediate risk* endometrioid EC after hysterectomy and bilateral salpingo-oophorectomy.	Experimental arm: molecular-integrated risk profile-based adjuvant treatment Standard arm: adjuvant vaginal brachytherapy	Vaginal recurrence	(46–48)
RAINBO program	- p53abn RED: III - MMRd-GREEN: III - NSMP-ORANGE: III - *POLE*mut-BLUE: II	Randomized, international	- p53abn-RED: 554 patients with invasive stage I–III p53abn EC; - MMRd-GREEN: 316 patients with stage II (with LVSI) or stage III MMRd EC; - NSMP-ORANGE trial: 600 patients with estrogen receptor positive stage II (with LVSI) or stage III no specific molecular profile (NSMP) EC - *POLE*mut-BLUE trial: 145 patients with stage I/III *POLE*mut EC	- p53abn-RED: adjuvant chemoradiation followed by olaparib for 2 years *vs* adjuvant chemoradiation alone; -MMRd-GREEN: adjuvant radiotherapy with concurrent and adjuvant durvalumab for 1 year *vs* radiotherapy alone; - NSMP-ORANGE: radiotherapy followed by progestin for 2 years *vs* adjuvant chemoradiation; - *POLE*mut-BLUE: no adjuvant therapy for lower-risk disease and no adjuvant therapy or radiotherapy alone for higher-risk disease.	p53abn-RED, MMRd-GREEN, and NSMP-ORANGE: RFS at 3 years; *POLE*mut-BLUE: pelvic recurrence at 3 years	(39, 49, 50)
CANSTAMP (recruiting)	II/III	Randomized, controlled, multi-arm, multi-stage, umbrella trial	267 women (estimated) with serous EC. Endometrioid and clear cell subtypes with abnormal/mutant- p53 are acceptable (PS ≤ 2). Stage I tumors will be enrolled in the early stage cohort. Life expectancy > 3 months.	- Early stage: EBRT *vs* vaginal brachytherapy after chemo in women with serous or p53-aberrant EC. -Advanced stage: Maintenance with experimental treatment (niraparib)	DFS at 3 years	(6, 51)
TAPER (recruiting)	II	Multicenter, single-arm, prospective cohort study	393 women (estimated) ≥ 18 years with stage I-III EC after hysterectomy and bilateral salpingo-oophorectomy (PS 0-2)	- Observation or Vaginal Brachytherapy - Interventions: Radiation; Vaginal brachytherapy; Other: Observation	Pelvic recurrence at 3 years	(6, 51)

*High-intermediate risk factors are defined as: (i) International Federation of Gynecology and Obstetrics stage IA (with invasion) and grade 3; (ii) stage IB grade 1 or 2 with age ≥60 and/or lymph-vascular space invasion; (iii) stage IB, grade 3 without lymph-vascular space invasion; or (iv) stage II (microscopic and grade 1).

DFS, Disease-free survival; EBRT, External beam radiotherapy; EC, Endometrial cancer; LVSI, Lymphovascular space invasion; MMRd, Mismatch repair deficiency; NSMP, No specific molecular profile; PS, Performance status.

POLE-mut EC showed favorable clinical outcomes, while retrospective studies revealed that avoiding adjuvant treatment is safe in this patient group. In addition, two prospective clinical trials, PORTEC-4 and TAPER, are underway to evaluate this aspect. The PORTEC-4a study will be the first to prospectively show the use of adjuvant therapy after combining molecular and clinic-pathological characteristics in EC (53). The RAINBO (Refining Adjuvant treatment iN endometrial cancer Based On molecular profile) study, instead, is an international TransPORTEC collaboration aimed at evaluating the molecular-based adjuvant treatment for high-risk EC women (6).

Furthermore, two additional studies carried out in Canada are examining specific molecular subtypes, such as the POLE and p53 status. The TAPER (Tailored Adjuvant therapy in POLE-mutated and p53 wildtype early-stage Endometrial cancer) is a multicenter, single-arm, prospective cohort study, which is specifically investigating treatment reductions in patients affected by early stage POLE-mutated/p53-wild-type EC with non-specific molecular profile (NSMP) (54). The second Canadian CANSTAMP trial (NCT04159155) is a randomized, controlled, multi-arm, multi-stage study evaluating the first-line treatment in serous or p53-mutated ECs (6, 55).

The finding of good clinical results in POLE-mut ECs highlights that this group of patients could be unnecessarily exposed to unwanted side effects, if subjected to radio- and/or chemotherapy (54). Therefore, the clinical practice, whereby many patients currently undergo adjuvant therapy, may represent an overtreatment, suggesting a reduction in treatment or perhaps no additional therapeutic option (19). Results from the ongoing PORTEC4a study will show whether this is a valid approach for patients with intermediate-risk POLE-mut EC. For high-risk POLE-mut ECs, the recently presented molecular characterization in the PORTEC-3 study is resulted highly informative (16, 56). POLE-mut tumors in the high-risk EC population showed an excellent prognosis when adjuvant treatment is restricted to radiotherapy, strongly suggesting that these patients do not benefit from adjuvant chemotherapy (16).

To date, treatment options for patients with advanced or recurrent EC are restricted. POLE-mut and dMMR ECs, due to their high mutational load, acquire high levels of neoantigens and TILs, making them interesting candidates for immunotherapy (57).

### 3.2 Immunotherapy as treatment option for metastatic and relapsed POLE-mutated EC

In general, EC women have a favorable prognosis, however patients with relapsed or metastatic disease have an overall survival (OS) poor (58). Emerging data suggests that knowledge of POLE status could be important in the context of relapse, as ultramutated tumors may be more likely to respond to immunotherapy (20, 59, 60).

Stasenko et al. (20) showed the effectiveness of immunotherapy agents in dMMR or MSI-H tumors. In particular, pembrolizumab, anti-PD-1 drug, has obtained the approval by the FDA for the treatment of these tumors. However, the role of immunotherapy in MSS tumors with POLE EDM and ultramutated phenotype is less clear (20, 61).

Although most POLE-mut ECs are MSS, preclinical data support the use of PD-1/PD-L1 inhibitors in this subgroup of ultramutated tumors, as the genomic instability present in tumors with a POLE EDM leads to a high number of neo-antigens and an increased quantity of TILs (62, 63). Several studies have reported the effective use of anti-PD-1 immune checkpoint inhibitors for the management of relapsed POLE-mutated EC (20, 64, 65).

Standard systemic treatment for metastatic disease includes hormone therapy for patients with low-grade, estrogen and progesterone receptor-positive tumors (66). Two randomized phase III clinical trials (KEYNOTE-775/NCT03517449 and ENGOT-EN9/LEAP-001/NCT03884101) focusing on the combinatorial use of lenvatinib and pembrolizumab compared to chemotherapy are currently recruiting (67, 68).

Finally, monotherapy with PARP and PD-1/PD-L1 inhibitors showed promising results (69, 70). Several phase II studies are evaluating whether combined therapy of PARP inhibition and PD-1/PD-L1 pathway inhibition is even more effective, as limited data have suggested an additive or even synergistic effect (69). The NCT02912572 study will include 70 patients with metastatic EC previously treated with at least one line of chemotherapy. The first cohort will include patients with dMMR and/or POLE mut EC treated with avelumab monotherapy. The second cohort will include patients with MSS without POLE mutation treated with either avelumab or talazoparib (58). The DOMEC study (NCT03951415), an ongoing multicenter, single-arm phase II study, will include 55 patients with metastatic EC to study the combination of olaparib and durvalumab, treatment response and recurrence-free survival (69, 71). **Table 2** summarizes the main studies involving the use of immunotherapy treatments currently used or under evaluation in POLE-mutated EC.

**Table 2:** Clinical trials involving immunotherapy treatments against POLE-mutated EC.

Trial name	Phase	Design	Patient population	Intervention arms	Primary endpoints	References
KEYNOTE-775 (NCT03517449)	III	Open-label, randomized, multicenter	827 women ≥ 18 years with advanced, recurrent or metastatic EC with radiographic evidence of DP after 1 prior systemic platinum-based chemo (PS = 0-1)	Experimental: Lenvatinib + Pembrolizumab; Active comparator: Paclitaxel/doxorubicin	PFS/OS	(72–74)
ENGOT-EN9/LEAP-001 (NCT03884101)	III	Randomized (1:1), Open-label, active-controlled trial	842 women ≥ 18 years with stage III/IV/recurrent EC measurable or radiographically apparent. Previous chemotherapy only as neoadjuvant/adjuvant therapy and/or concurrently with radiation.	Experimental: Lenvatinib + Pembrolizumab; Active comparator: Paclitaxel + carboplatin	PFS/OS	(67, 75, 76)
NCT02912572 (active, not recruiting)	II	Two-Group, Two-Stage, Open-Label Study	106 women ≥ 18 years with MSS, MSI-H and POLE-mutated recurrent or persistent EC.	Experimental: POLE- Mutated EC (avelumab); Experimental: MSS EC (avelumab); Experimental: MSS EC (avelumab/talazoparib combination arm); Experimental: MSS EC (avelumab/axitinib combination arm)	ORR/ PFS at 6 months	(77, 78)
DOMEC (NCT03951415)	II	Prospective, multicenter study	55 women > 18 years with advanced (recurrent, refractory or metastatic) EC or carcinosarcoma of the endometrium.	Experimental: PARP inhibitor and Anti-PD-L1	PFS at 6 months	(71, 79, 80)

DP, Disease progression; EC, Endometrial cancer; MSI-H, microsatellite instability high; MSS, microsatellite stability; ORR, Objective Response Rate; OS, Overall Survival; PFS, Progression-Free Survival.

Ionizing radiations have been shown to induce genomic instability through direct and indirect damage to DNA, resulting in ROS production, which triggers chemical reactions with potentially lethal damage (81). The combination of radiotherapy with PARP inhibitors leads to the inhibition of DNA repair mechanisms in malignant cells, which contributes to promote the DNA damage and apoptosis (82, 83). Furthermore, radiations enhance tumor immunogenicity through the release of proinflammatory cytokines and chemokines via tumor antigen uptake and cross-presentation by dendritic cells (84). The combination of immunotherapy and radiations determines an alteration of the tumor microenvironment by intensifying the recruitment and infiltration of immune cells (58).

## 4 Prevention pathway: genetic counseling and screening

Genetic counseling is a procedure that analyzes personal and family history of patients to evaluate the presence of hereditary features. The objective of the genetic counseling is to identify potential correlations between the risk of developing a hereditary cancer syndrome and the presence of an underlying predisposing mutation, in order to increase awareness and possibility of adopting suitable screening strategies (13, 85).

Somatic POLE alterations have been found in 6-10% of ECs, but germline *POLE* PVs may also predispose to EC. However, germline *POLE* alterations in this tumor are rare, since they have been reported only in 0.25-4% of EC cases (13). Several reports support the hypothesis that germline PVs in *POLE* (exons 9-14) and *POLD1* (exons 8-13), which are deleterious to polymerase proofreading function, predispose to EC (86–89). However, to date, due to the paucity of data in this regard, the risks of EC associated with POLD1/POLE PVs have yet to be estimated. Furthermore, though little is known about prognosis of germline PV carriers, somatic *POLE* alterations correlate with favorable clinical outcome in EC women.

Although the absolute risk of EC associated with germline *POLD1*/*POLE* PVs has not been established, Bellido et al. (89) published preliminary clinical management recommendations, suggesting a colonoscopy every 1–2 years and gastroduodenoscopy every 3 years, starting at the age of 20–25 years (the periodicity should be re-evaluated according to the findings), adding EC screening beginning at the age of 40 years for female carriers of germline *POLD1* PVs.

The germline *POLE*/*POLD1* genetic testing should be considered in the context of a strong family history of colorectal or endometrial cancer (two or more relatives) or a single relative with colorectal cancer or endometrial cancer <60 years (89).

The advent of new techniques, such as NGS, provided substantial advantages compared to single-gene testing. Therefore, cancer-related multiple genes should be tested in patients with suspect POLE associated tumor syndromes (13).

Although *POLD1* and *POLE* have recently been suggested as genes predisposing to hereditary EC, absolute risk for EC and even other cancer types is very uncertain, therefore the *POLD1*/*POLE* genetic testing of unselected EC patients should be restricted to the research setting (90).

## 5 Discussion

Somatic POLE mutations have been detected in 6-10% of EC cases, whereas germline POLE alterations are very rare (0.25-4%), limiting the applicability of findings to a broader EC population (13). Although germline POLE genetic testing can enable the inclusion of mutation carriers in suitable and intensive preventive and surveillance pathways, its performing involves ethical and psychological implications for probands and family members, causing a state of potential anxiety or misinterpretation of cancer risk (90). Additionally, not all POLE mutations have an established clinical significance, as some variants of unknown significance (VUS) may complicate diagnosis and treatment decisions. Functional studies are often required to confirm pathogenicity (91). Another issue regards the access to comprehensive genetic testing (somatic and germline), which may not be uniformly available, increasing the variability (92). Lack of standardized protocols for testing and interpreting POLE variants could lead to inconsistent clinical application (91, 93). Despite data on long-term outcomes in germline mutation carriers are still limited (13), however, some evidence supports the integration of the routine POLE mutation testing in EC patients, especially useful for stratifying treatment and managing familial cancer risk.

POLE EDMs identify a subgroup of EC patients with an excellent prognosis. The use of this biomarker has been suggested to refine adjuvant treatment decisions, also helping to identify patients who would benefit from immune checkpoint inhibitors (59).

**Figure 3:**
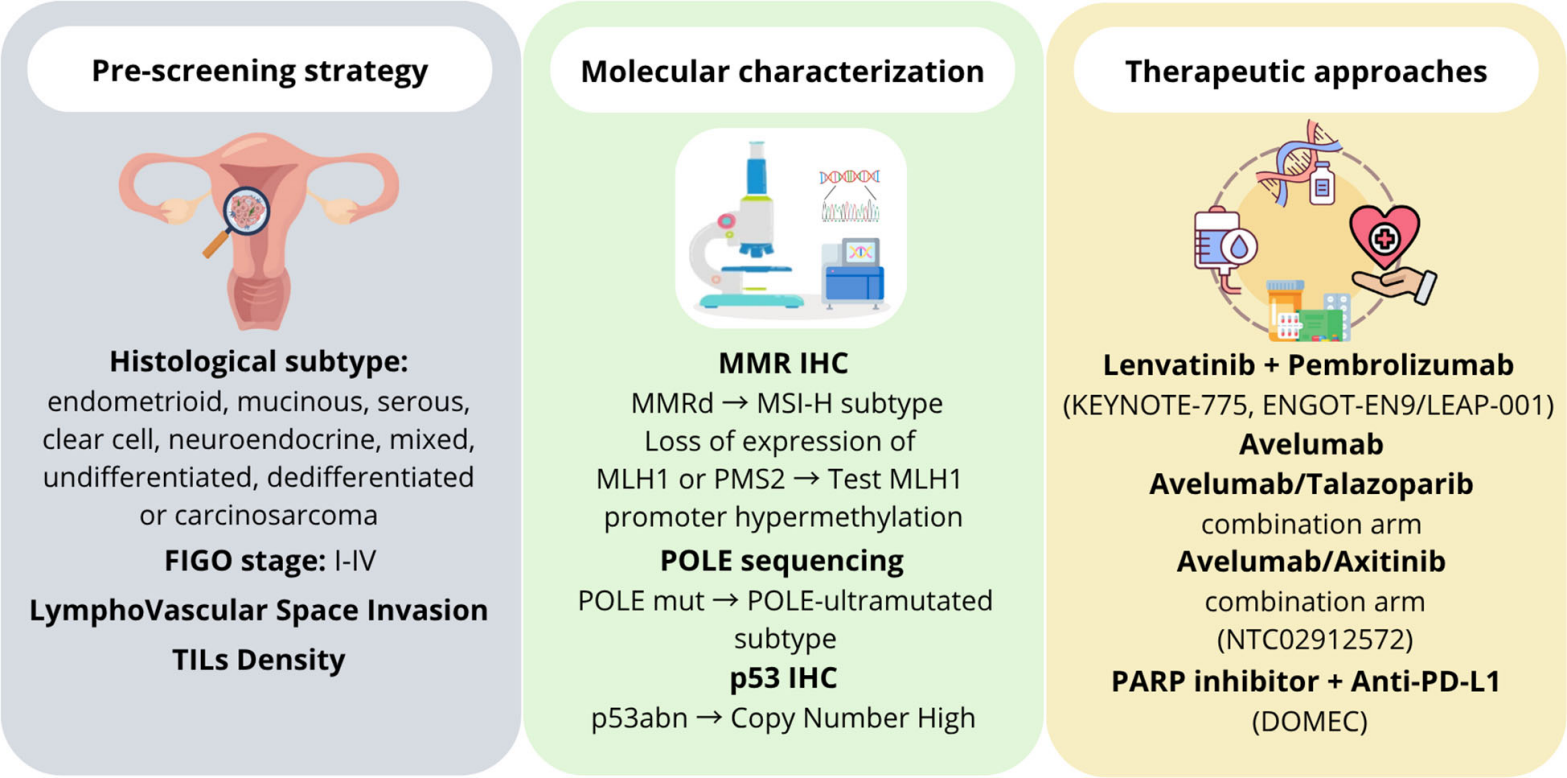
Schematic representation of theranostic approaches currently used in POLE-mutated endometrial cancer. The left panel shows the pre-screening strategies mainly based on morphological and histological features of endometrial cancer. The middle panel reports the approaches used for the molecular characterization. The right panel lists the main therapeutic strategies currently used or under evaluation against POLE-mutated endometrial cancer.

Despite high tumor grade, POLE-ultramutated ECs showed excellent clinical outcomes, strong immune response, potentially higher sensitivity to immunotherapy (20, 94, 95). The screening for *POLE* alterations is performed by sequencing the exonuclease domain (exons 9-14), but the relatively high cost of this analysis hinders the clinical use of this important biomarker. The evaluation of simple morphological, histological and immunohistochemical features (tumor type, grade, peritumoral lymphocytes, MLH1 and p53 expression) could help in the pre-screening phase and molecular characterization for identifying POLE EDMs in EC, increasing the likelihood from 7% to 33% that a mutation may be detected (15) (**Figure 3**, left and middle panels). Different therapeutic strategies are currently used or under evaluation for encouraging the development of targeted therapies and personalized treatments in women affected by POLE-mutated EC (60) (**Figure 3**, right panel). This could facilitate the use of this significant prognostic and predictive biomarker in routine clinical practice (96). Although POLE-ultramutated ECs show immune activation, not all cases respond to immune checkpoint inhibitors, as immune microenvironment and co-mutations may influence therapeutic outcomes (97, 98). Therefore, a potential overestimation of immunotherapy benefit is a problem that clinicians should take into account. Finally, most data supporting POLE as a biomarker are from retrospective studies or small cohorts. More prospective clinical trials are needed to validate POLE status as a predictor of immunotherapy response (92).
